# A possible volcanic origin for the Greenland ice core Pt anomaly near the Bølling-Allerød/Younger Dryas boundary

**DOI:** 10.1371/journal.pone.0331811

**Published:** 2025-09-18

**Authors:** Charlotte E. Green, James U. L. Baldini, Richard J. Brown, Hans-Ulrich Schmincke, Marie Edmonds, Thomas C. Meisel

**Affiliations:** 1 Centre of Climate, Ocean and Atmosphere, Department of Earth Sciences, Royal Holloway, University of London, Egham, United Kingdom; 2 Department of Earth Sciences, University of Durham, Durham, United Kingdom; 3 GEOMAR Helmholtz Centre for Ocean Research, Kiel, Germany; 4 Department of Earth Sciences, University of Cambridge, Cambridge, United Kingdom; 5 General and Analytical Chemistry, Technical University of Leoben, Leoben, Austria; Maria Curie-Sklodowska University: Uniwersytet Marii Curie-Sklodowskiej, POLAND

## Abstract

The Younger Dryas Event (YDE) is the most recent and most well-understood millennial-scale cooling event. A deglacial meltwater pulse is the traditionally accepted trigger for the event, but both a bolide impact and volcanism are recently advanced alternative explanations. A high Pt/Ir and Pt/Al geochemical anomaly within the Greenland Ice Sheet Project (GISP2) ice core, broadly coinciding with the YDE initiation, provides a possible geochemical clue to the events leading up to the YDE. Previous research has suggested that the impact of an unknown type of high Pt/low Ir iron meteorite may have produced this Pt spike, but the timing is also very close to a large sulphur spike within the North Greenland Ice Core Project (NGRIP) ice core and the timing of the Laacher See volcano eruption (which occurred at approximately 13 ka), suggesting a possible volcanic origin. Here, we evaluate both suggestions by i) presenting new geochemical data from the Laacher See Tephra (LST) and ii) confirming the Pt spike timing relative to the YDE onset on the GICC05 timescale. Our geochemical results, and specifically iridium and platinum data, strongly suggest that the Laacher See eruption (LSE) was most likely not the source of the Greenland Pt spike. Additionally, we corroborate recent work showing a chronological offset of several decades between the Pt spike and the North Greenland Ice Core Project (NGRIP) sulphur spike, the initiation of the YDE at 12,870 ± 30 yr BP (years before present, where present is defined as 1950 CE), and the nearest published age estimate for the LSE (12,880 ± 40 yr BP – though we note that more recent age determinations potentially push this date back by ~130 years). Based on modern data showing that Pt spikes in ice cores and sediment can arise from volcanic eruptions, we suggest that the GISP2 Pt anomaly may represent fractionated volcanic material from another, unknown volcanic eruption. Volcanic gas condensates from submarine volcanic complexes, and in particular Niuatahi-Motutahi (Tonga rear arc), have a Platinum Group Element (PGE) geochemistry most resembling the Pt spike, and we therefore suggest that the Pt spike represents highly fractionated material from an Icelandic subglacial or submarine fissure eruption. The 14-year-long duration of the Pt spike is also more consistent with a fissure eruption than an instantaneous event.

## Introduction

The Younger Dryas (YD) climate anomaly [1] was characterised by an abrupt return to cold and dry stadial conditions across the North Atlantic and Europe following the warm and wet conditions of the Bølling-Allerød interstadial. Lower oxygen isotope ratios (δ^18^O) in the Greenland Ice Sheet (GIS) across this period (from ~12,870 to 11,700 yr BP, termed Greenland Stadial-1, or ‘GS-1’) reflect not only lower temperatures, but also increasing Arctic sea ice extent and altered moisture mass trajectories [1–3]. GS-1 is now widely believed to reflect the entire North Atlantic cooling episode, whereas the YD may reflect the response in northern Europe, either delayed by ~130 years [4–7] or occurring simultaneously relative to Greenland cooling [8–10]. However, broad agreement exists that GS-1 and the YD are manifestations of the same climate anomaly, referred to here as the ‘Younger Dryas Event’ (YDE) for simplicity.

A consensus also exists that a reduction in North Atlantic deep-water (NADW) formation [11,12] coupled with sea ice growth explains much of the YDE’s climate signature, but the trigger of this ocean circulation reorganisation is still poorly understood and controversial [13,14]. The most well-researched hypothesis invokes a meltwater pulse from a large glacial lake (e.g., Lake Agassiz) formed during Laurentide Ice Sheet decay, which caused Atlantic Meridional Overturning Circulation weakening by halting NADW formation in the northern North Atlantic [e.g., 15, 16–21]. However, despite decades of research, the source and pathway of the meltwater is still ambiguous [15,18,22–24].

An alternative hypothesis, initially proposed by Firestone et al. [25], suggested that one or more bolides struck or exploded over the Laurentide Ice Sheet at ~12,900 yr BP, a hypothesis referred to as the ‘Younger Dryas Impact Hypothesis’ (YDIH). Evidence that has been interpreted to support this hypothesis comes from terrestrial sediments, lake cores, and ice cores [26–30]. However, competing alternative explanations for the same geological evidence were advanced [31–36], and the physics of the proposed impact event was questioned [37]. Additionally, the presence of YDE-like events during older deglacial intervals [13] and of numerous topologically similar stadial events across the last glacial [38] suggest potentially commonplace mechanisms were responsible, rather than a catastrophic extraterrestrial trigger. A comprehensive catalogue of potential issues with the YDIH was recently reported by Holliday et al. [39].

Petaev et al. [40] described an interval of the Greenland Ice Sheet Project (GISP2) ice core that contained high platinum (Pt) concentrations and broadly coincided with the YDE onset. The high Pt/iridium (Ir) and Pt/aluminum (Al) ratios are difficult to reconcile with known materials, and therefore Petaev et al. [40] suggested that the Pt anomaly could represent an unusual, highly differentiated, Ir-poor iron meteorite, but the precise nature of the possible impactor remained enigmatic. Subsequently, Wolbach et al. [30] presented 26 YDE boundary sites with high Pt concentrations within sediment, some with other potential impact-related proxies, and attributed the anomaly to a bolide impact that triggered the YDE.

**Fig 1:**
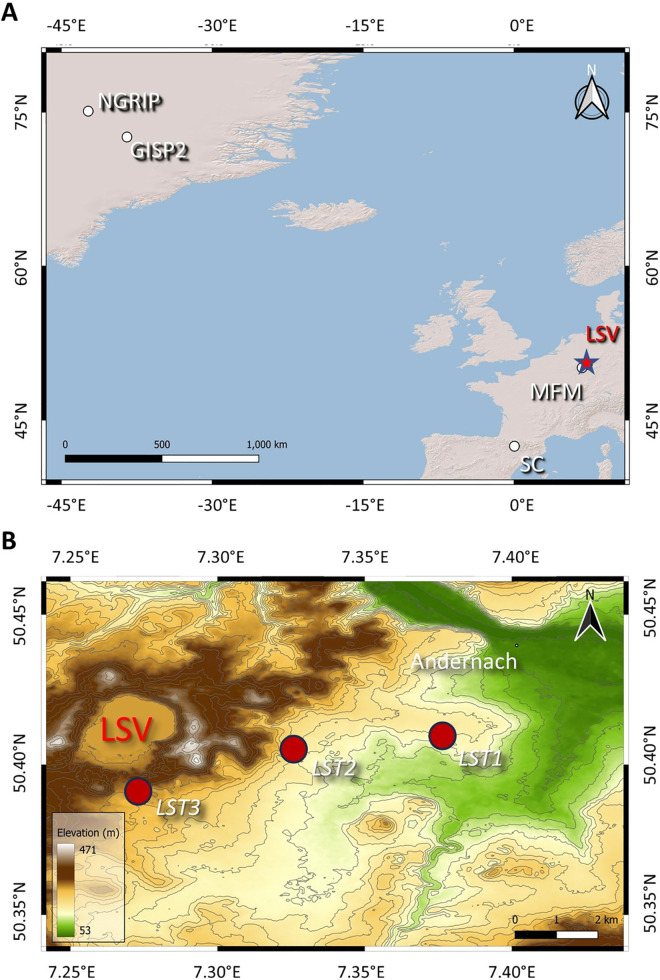
Site locations. A) Locations of sites referred to in the text on map of the North Atlantic region: Laacher See Volcano (LSV), NGRIP, GISP2, Seso Cave (SC), and Meerfelder Maar (MFM). Basemap: Esri World Shaded Relief (Source: Esri, ©2025). B).Location of the three tephra sampling locations on Digital Elevation Map of Laacher See area. Shown on map are the Laacher See volcano (LSV), the nearest town (Andernach), LST1 (50°24’38” N, 7°22’31” E, Lower Laacher See Tephra), LST2 (50°24’20 “N, 7°19’31.63”E, Middle Laacher See Tephra), and LST3 (50°23’30” N, 7°16’23” E, Upper Laacher See Tephra). Further details provided in Supporting Information. Digital Elevation Model (DEM) with 20 m interval contours derived from NASA’s Shuttle Radar Topography Mission (SRTM) 1 Arc-Second Global dataset (∼30 m resolution), downloaded from USGS Earth Explorer. Elevation is referenced to the EGM96 vertical datum, with horizontal coordinates in geographic (WGS84) projection. Scale bar represents two kilometres.

A third hypothesis is that volcanism was responsible for triggering the event [41,42], as was suggested for other stadials [43]. A candidate eruption is the Laacher See eruption (LSE) in the East Eifel Volcanic Field, Germany (Fig 1), which has been proposed as contributing to the rapid climate shifts associated with the YDE [16,42,44]. The LSE had a Volcanic Explosivity Index of 6, erupted 6.3 km^3^ (dense rock equivalent) of chemically-zoned phonolite in predominantly fallout deposits, released 3–15 Tg sulphur [45], and was up to twice the size of the 1991 CE Pinatubo eruption [44,46–48].

A large volcanic sulphate spike exists at ~12,870 yr BP within the NGRIP ice core and other cores from both Greenland and Antarctica [41,49]. Whether the eruption responsible for the ~ 12,870 yr BP spike was the Laacher See volcano eruption is the subject of considerable debate. Brauer et al. [50] used lake varves to date the LSE to 12,880 ± 40 yr BP; indistinguishable from the timing of the large volcanic sulphate spike. This age is also most consistent with an argon-argon age for the eruption of 12,900 ± 560 yr BP [51], though this date does have very large uncertainties.

Two new high-profile ages challenge this perspective, with a newer age determination for the LSE of 13,006 ± 9 yr BP published by Reinig et al. [8], based on high-resolution radiocarbon measurements of subfossil trees that were buried in pyroclastic density current deposits. However, a recently published comment raises the possibility that the date was affected by radiocarbon ‘dead’ magmatic carbon dioxide [6], which was not accounted for, though this suggestion was countered by Reinig et al [52]. The Reinig age is also supported by a recent U-Th-based age determination dating the inferred geochemical signal of the eruption within a German stalagmite [9]. The choice of LSE date does not affect our main conclusions regarding the origin of the platinum spike discussed below, which are based on the presence of a volcanic sulphate spike within Greenland ice cores rather than the timing of a specific eruption.

Wolbach et al. [30] briefly consider volcanism as the Pt anomaly’s origin, but ultimately discarded the idea because previous research [25,28] detected no volcanic tephra in North American terrestrial samples. However, the Pt anomaly’s age broadly coincides with the Brauer et al. [53] LSE date, and, given the eruption’s unusual phonolitic composition, it is conceivable that the eruption was the source of the Greenland Pt anomaly [42]. No tephra from the LSE has been identified in the Greenland ice cores, suggesting that it is unlikely that the tephra would exist in North American sediment sequences. Therefore, not finding the tephra in the sequences as suggested by Wolbach et al [29] is not a reason for disregarding the eruption, or indeed volcanism in general, as a cause for the YDE. Furthermore, the refined age of 12,870 ± 30 yr BP for the YDE onset in the North Atlantic [54] coincides with the timing of the recently reported S spike in the NGRIP ice core [41], which provides very clear evidence that a large NH volcanic eruption did occur then.

However, whether the LSE was enriched in Pt is currently unknown, though other eruptions have resulted in Pt enrichment in glacial ice. Data from Greenland ice cores illustrate that the 1991 CE eruption of nearby Hekla volcano (Iceland), and potentially even the distal Mount Pinatubo (Philippines), caused Pt anomalies [55]. Similarly, Soyol-Erdene et al. [56] described Pt spikes in Antarctic snow linked to several 20^th^ Century volcanic eruptions, the most prominent peak coinciding with the 1991 Cerro Hudson (Chile) eruption. Despite firm evidence that volcanism can cause Pt concentration spikes in ice cores and terrestrial sediments, volcanism as an explanation for the Pt anomaly in the GISP2 ice core has not been thoroughly considered, with most researchers instead opting to ascribe the anomaly to the impact of an unknown type of bolide.

Here, we independently evaluate recent analyses of the GISP2 Pt spike’s timing relative to the LSE and the YDE utilising the GICC05 chronology [39,41,57] and apply this information to reconstruct a detailed series of events leading up to the YDE. We also present new geochemical data for the Laacher See Tephra to evaluate whether the Pt spike in the GISP2 ice core could have resulted from the LSE. Additionally, we compare the geochemical ratios of the LST, the GISP2 Pt spike, and published values of extraterrestrial and volcanogenic rocks and sediments to determine if the Pt anomaly could reflect the LSE, another unknown eruption, or an extraterrestrial impact.

## Materials and methods

### Geochemical analyses of the Laacher See Tephra

We obtained 17 samples from the Lower Laacher See tephra (LLST), the Middle Laacher See tephra (MLST), and the Upper Laacher See tephra (ULST) units of the Laacher See Tephra at three localities (LST1, LST2 and LST3; see Fig 1 and S1 Table) comprising samples of pumice only, lithic clasts only, or both (“bulk” samples). A topsoil sample from locality LST1 was analysed to assess contamination in the soil from catalytic converters on automobiles. The individual elements Pt, Ir, Hf, Lu, and Al were also quantified (S2 Table) because these data exist across the Greenland Pt anomaly [40], facilitating intercomparison. Geochemical ratios were used rather than absolute abundances because of concentration differences between sources and ice sheet are likely considerable. Values were normalised to chondrite values [58] to allow comparison between extraterrestrial and terrestrial materials.

Samples were obtained from three localities within 10 km of the Laacher See volcano directly from outcrops of the Laacher See Tephra from the ~ 13,000 yr BP eruption. We excavated approximately 10–20 cm into the outcrop to remove potentially contaminated surface tephra, then picked individual pumice clasts with few to zero visible lithics. To ensure that no Pt-rich horizon was missed, the samples were selected from material released across the entire eruption. In total, 18 sample bags (17 with pumice/lithics/bulk samples + 1 with topsoil) were filled from the lower LLST, MLST, and ULST units. The samples collected were named: LLST: LST001, LST001A, LST002, LST002A, LST003, LST003A and LST003B; MLST: LST004, LST005, LST006, LST007 and LST007A and ULST: LST008 (see SOM). Bulk samples and samples of lithics, pumice, and one sample of topsoil from the LLST outcrop were also collected.

### Sample preparation and analysis

Throughout sample collection and preparation, care was taken to avoid contamination. The samples were soaked for approximately two hours in glass beakers filled with deionised water and rinsed with deionised water in the laboratory. The samples were left to dry over several days before overnight drying in a laboratory oven at 70°C. Nitrile gloves were used to handle the clean samples. Whole rock analysis was conducted at ActLabs, who crushed and powdered the samples. Smaller samples were pulverised to 95% passing through a 74 μm sieve. The topsoil sample (LST1 TS) was dried to 60°C and sieved.

Fire assay and inductively coupled plasma mass spectrometry (ICP-MS) methods were used to analyse whole rock samples for Pt, using a Perkin Elmer Sciex ELAN ICP-MS. The detection limit was 0.1 ppb, determined by ActLabs via repeat blank analyses. To analyse whole rock samples for Ir, a nickel sulphide (NiS) fire assay procedure and instrumental neutron activation analysis (INAA) technique was utilised after Hoffman [59].The detection limit was 0.1 ppb. Concentrations for elements that are below detection limits are shown in plots at the detection limits; this only affects values plotted in Fig 4, and further details are provided in the caption. This does not affect the interpretations or conclusions. A combination of lithium metaborate/tetraborate fusion applied to ICP-OES for whole rock major element and ICP-MS for trace element concentration quantification was utilised, using a Perkin Elmer Sciex ELAN ICP-MS for analysis. Standards utilised included SY-4, W-2a, ZW-C, NCS DC86316, NCS DC86318, USZ 44−2007, and REE-1. The precisions on standard reference materials for Pt, Ir, Al, Hf, and Lu measurement results were <4% for Pt, < 5% for Ir, Hf and Lu and <3% for Al.

**Fig 2:**
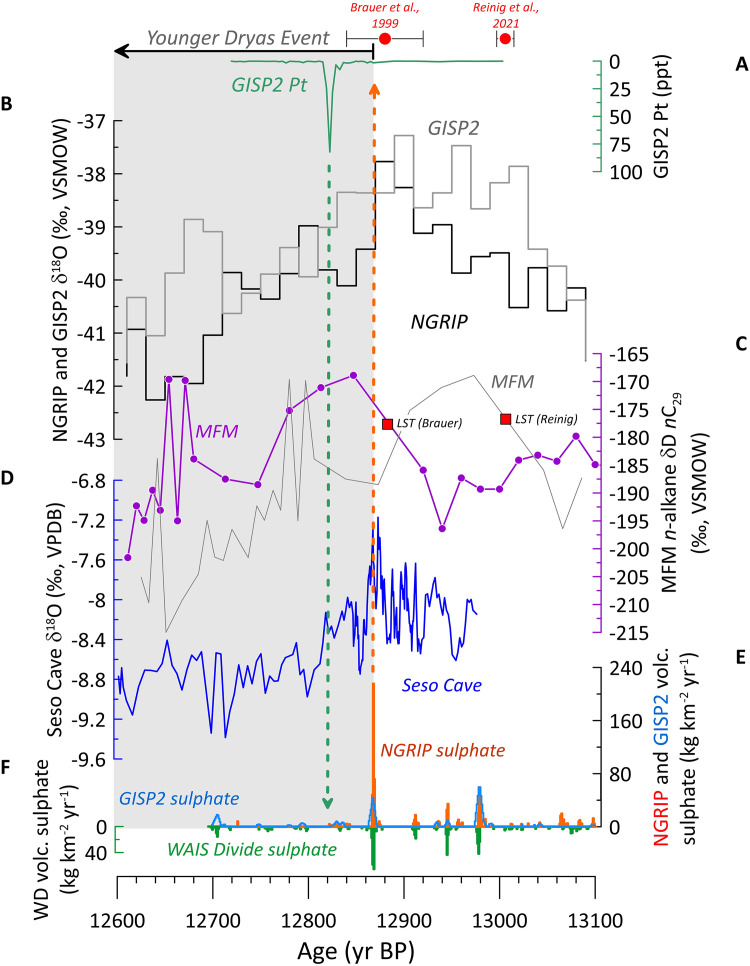
Key datasets. Greenland and European YDE datasets. A) Platinum concentrations in the GISP2 ice core [40]; B) Bidecadally-resolved GISP2 and NGRIP δ^18^O [49]; C) MFM hydrogen isotope values (δD) recorded in *n*-alkanes of terrestrial lipids (‰) [114], shown using the Brauer et al [50] (purple with circles) and Reinig et al [8] (grey) ages as chronological controls (red squares indicate the timing of the LST in the records). The choice of date does not affect other records shown, which do not contain the LST. D) Seso Cave δ^18^O record [54]; E) Volcanic SO_4_ in the NGRIP (orange) and GISP2 (blue) ice cores [8,41]. F) Volcanic SO_4_ in the WAIS Divide (WD) ice core [8,41]. The grey box represents part of the YDE from its onset 12,870 ± 30 yr BP [54] to 12,600 yr BP. The two most widely used high-precision dates for the LSE are shown at the top (red circles). The vertical dashed arrows represent the timing of the 12,871 yr BP NGRIP sulphur spike (red) and the timing of the 12,822 yr BP GISP2 platinum spike (green).

**Fig 3:**
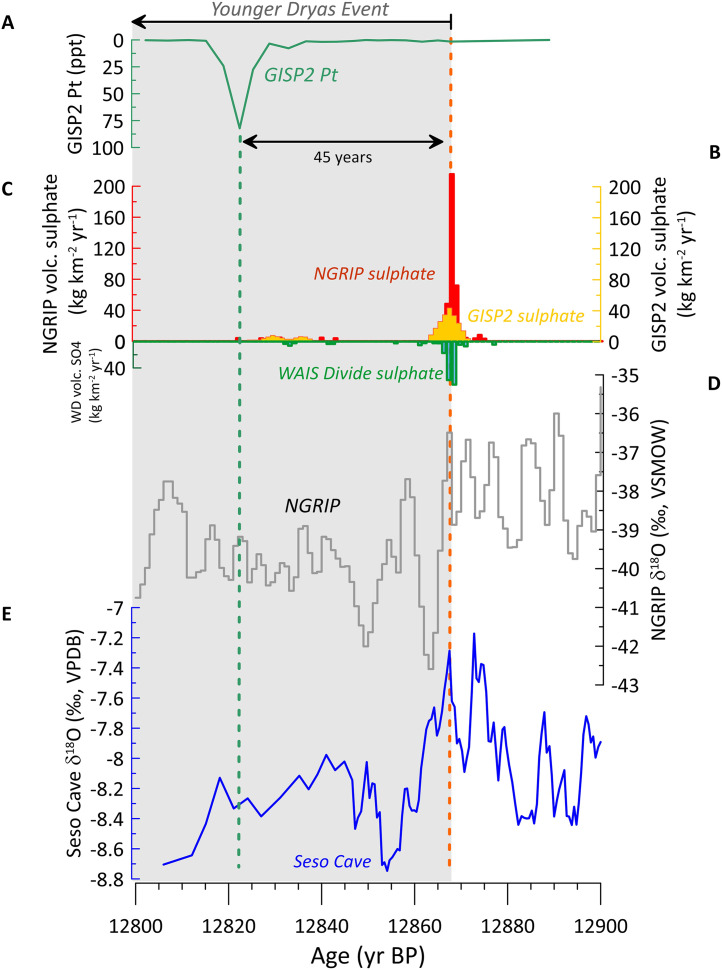
An expanded view of key records covering the YD/BA transition. A) Platinum concentrations in the GISP2 ice core [40]; B) Volcanic sulphate in the GISP2 ice core (yellow) [115]; C) Volcanic sulphate in the NGRIP ice core measured by discrete sampling (red) [41] and WAIS Divide (WD) ice core volcanic sulphate (green) [8,41]; D) Annually-resolved NGRIP δ^18^O [64]; E) Seso Cave δ^18^O record [54]; The grey box represents part of the YDE from its onset 12,870 ± 30 yr BP [54] to 12,800 yr BP. As in Fig 3, the vertical dashed arrows represent the timing of the 12,871 yr BP NGRIP sulphur spike (red) and the timing of the 12,822 yr BP GISP2 platinum spike (green).

## Results

### Timing of events

The GISP2 ice core Pt anomaly timing is compared here to volcanic sulphate in the West Antarctic Ice Sheet (WAIS) Divide, the GISP2, and NGRIP ice cores, bidecadal δ^18^O in the NGRIP and GISP2 ice cores, δ^18^O in Seso Cave (Spain) stalagmites, and to Meerfelder Maar (MFM) (Germany) varved lake core *n*-alkane records (Fig 2). The sulphate spike within GISP2 previously identified as potentially reflecting the LSE occurs at 12,867 ± 136 yr BP [42], and probably reflects the same event as a very large spike within the NGRIP core at 12,871 ± 136 yr BP [41,60] using discrete sampling and 12,867 yr BP using continuous flow analysis [61]; here we refer to this event as the ~ 12,870 years BP event. Mercury (Hg) is an element often associated with volcanic eruptions [62], and the largest peak in Hg concentrations in the EGRIP ice core across the deglacial interval (from 15,700 to 11,000 yr BP) occurs at ~12,870 yr BP [63], suggesting that a very large eruption occurred then (Fig 3), though it may also reflect environmental changes associated with the transition to the YDE. NGRIP ice core δ^18^O values decrease immediately after the spike at ~12,870 yr BP (Figs 2 and 3), which is consistent with the high resolution Seso Cave (Spain) stalagmite δ^18^O record that places the YDE onset in the North Atlantic at 12,870 ± 30 yr BP [54]. However, the Pt anomaly occurs ~45 years after the candidate sulphate spikes and the initial decline in ice δ^18^O, and so is not directly linked to either the start of the YDE or the sulphate spike at ~12,870 yr BP. The results above independently confirm the analysis previously reported by Svensson et al [57], Abbott et al [41], and Holliday et al [39] that the Pt spike post-dates the initiation of the YDE by several decades.

Comparison of the annual NGRIP δ^18^O record [64], the Seso Cave δ^18^O record, volcanic sulphate in the WAIS Divide (Antarctica) and NGRIP (Greenland) ice cores, and the GISP2 Pt concentrations yield even more detailed insights (Fig 3). The annual NGRIP δ^18^O record confirms that the YDE onset preceded the GISP2 Pt spike by 45 years, consistent with previous results [39,41,57]. Furthermore, the timing of the large volcanic sulphate spike in the NGRIP core (Figs 2 and 3) is indistinguishable from the onset of climate deterioration into the YDE apparent in both the NGRIP and Seso Cave δ^18^O records (Fig 3).

### Laacher See Tephra geochemistry

Platinum concentrations are low in the LST (generally near or below the 0.1 mg/kg detection limit). The highest mean concentrations occur in the basal LLST unit (0.2 mg/kg), and concentrations decrease upwards through the deposit until falling below detection limit (bdl) in the ULST. Iridium concentrations are below detection limits in all the LST pumice samples. Lutetium (Lu) and Hf concentrations are well above their respective detection limits (0.01 mg/kg and 0.2 mg/kg, respectively), and their concentrations decrease from the deposit base (the LLST) to the ULST. Hafnium shows a particularly strong decreasing trend from ~34 mg/kg to ~5 mg/kg. The Hf/Lu, Pt/Lu, Hf/Al, and Lu/Al ratios decrease upward from the basal LLST to the ULST, whereas the ratios Pt/Al and Pt/Hf decrease from the LLST to the MLST. The Laacher See topsoil sample has a higher Pt concentration (0.9 mg/kg), potentially due to modern anthropogenic contamination, but the very low LST values (generally below the detection limit) indicate that modern contamination of the LST pumice did not occur.

## Discussion

### Timing of the GISP2 Pt anomaly

Petaev et al. [40] measured Pt concentrations in the GISP2 ice core across the depths 1719.875 to 1709.000 m, representing the age range ~13,000 to 12,720 yr BP on the GICC05 timescale. The mean Pt concentration across this interval is ~ 3.6 ppt, and the maximum Pt concentration (the Pt ‘anomaly’) at 12,822 yr BP is 82.2 ppt (Figs 2 and 3) (note that values in tephra are in mg/kg and in ice are in ppt, and that therefore they are not directly comparable). Elevated Pt concentrations (between ~8–28 ppt) compared with surrounding ice also occur across the age range 12,833–12,819 yr BP (Figs 2 and 3). It is worth noting that Pt ions are potentially mobile when complexed as a halogeno or sulphato complex, but existing studies of volcanic Pt signals in glacial ice (e.g., [55]) suggest that Pt peaks within ice are sharp and distinct. Pt mobility within glacial ice therefore appears limited and cannot explain the several decades between the volcanic sulphate peak at the YDE onset and the later Pt peak. The fact that elevated Pt concentrations exist across the interval from 12,833 to 12,819 years suggests a persistent yet variable flux of Pt lasting about 14 years, rather than a short-lived event like a single explosive eruption or a bolide impact.

The GISP2 Pt spike timing was initially reported on the Meese-Sowers depth-age timescale; below we discuss the timing using the more recent GICC05 depth-age timescale synchronizing the GRIP, NGRIP, and GISP2 ice cores [1,49]. During the preparation of this manuscript, several other researchers have reported on a similar exercise. Svensson et al [57], Abbott et al. [41], and Holliday et al [39], all reported on their work converting the timing of the Pt spike to GICC05 depth-age timescale and comparing this to the YDE onset. Our work was conducted independently and in parallel with these works, without any knowledge of their results (see also our original work [65]), so there is value in reporting our results as an independent test of this previous research rather than simply citing these previously published results. Our results confirm the conclusions of these other works, that the Pt spike occurs several decades after the onset of the YDE, so whatever caused the Pt spike occurred too late to be a causal mechanism of the YDE.

### Geochemical constraints on the origin of the GISP2 Pt anomaly

**Fig 4:**
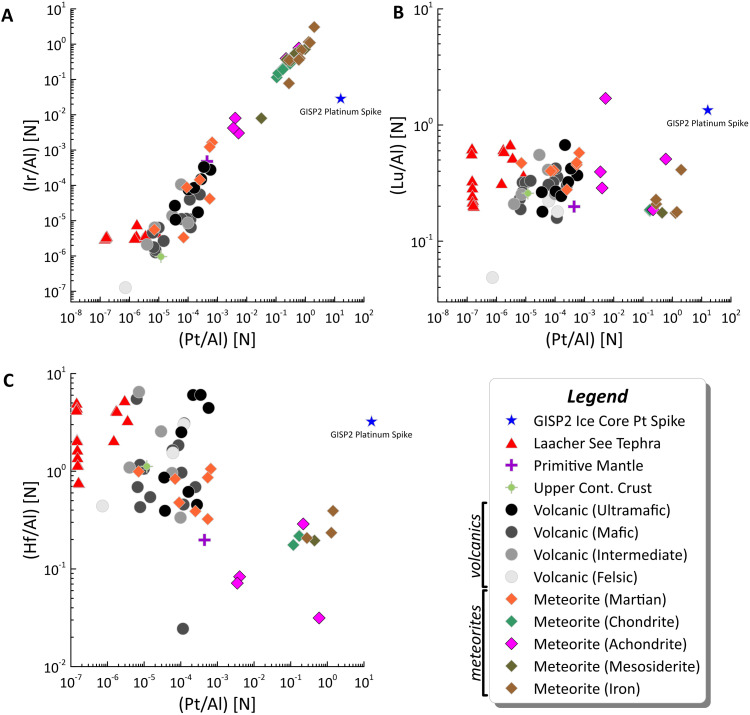
Geochemistry of relevant primary materials. Pt/Al ratios against (A) Ir/Al, (B) Lu/Al, and (C) Hf/Al ratios of the GISP2 spike, LST, volcanics and meteorites all normalised [N] to the respective ratio of chondrite [58]. Propagated errors on Pt/Al, Ir/Al, Hf/Al and Lu/Al are all < 5% and are smaller than the symbols used in the figure. Some of the LST Pt and Ir concentrations are below the detection limits; rather than not show these points we have plotted them at the detection limits to reflect the maximum possible ratio. References are provided in the SOM.

The results of this study show that, despite its unusual geochemistry, the LST is Pt-poor and below the crustal abundance of 0.4 μg/kg [66]. The LST and Pt spike data were compared to published values for volcanic deposits, meteorites, and selected sedimentary deposits. The LST samples mostly plot outside of the published volcanic field, although there is some minor overlap in Ir/Al against Pt/Al ratios (Fig 4). Elemental ratios in the LST are low in respect to other materials, often two orders of magnitude smaller than published volcanic values. Differences between the volatile concentrations and those remaining in the rock, measured here, might account for some of the observed differences. Regardless, the geochemical and chronological results presented here very strongly suggest that the LSE was not responsible for the GISP2 Pt anomaly.

The geochemical excursion within the GISP2 ice core is also dissimilar to any known meteorites or volcanic materials in terms of many elemental ratios: Hf/Al, Lu/Al, and Ir/Al against Pt/Al (Fig 4). For Ir/Al vs Pt/Al, the GISP2 spike plots closest to the meteorite field, although GISP2 Pt/Al values are still orders of magnitude larger than published meteorite values (at least ~ 10x, with some values ~ 100x), illustrating the unusually high Pt concentrations of the ice samples compared to Ir, Hf, and Lu.

**Fig 5:**
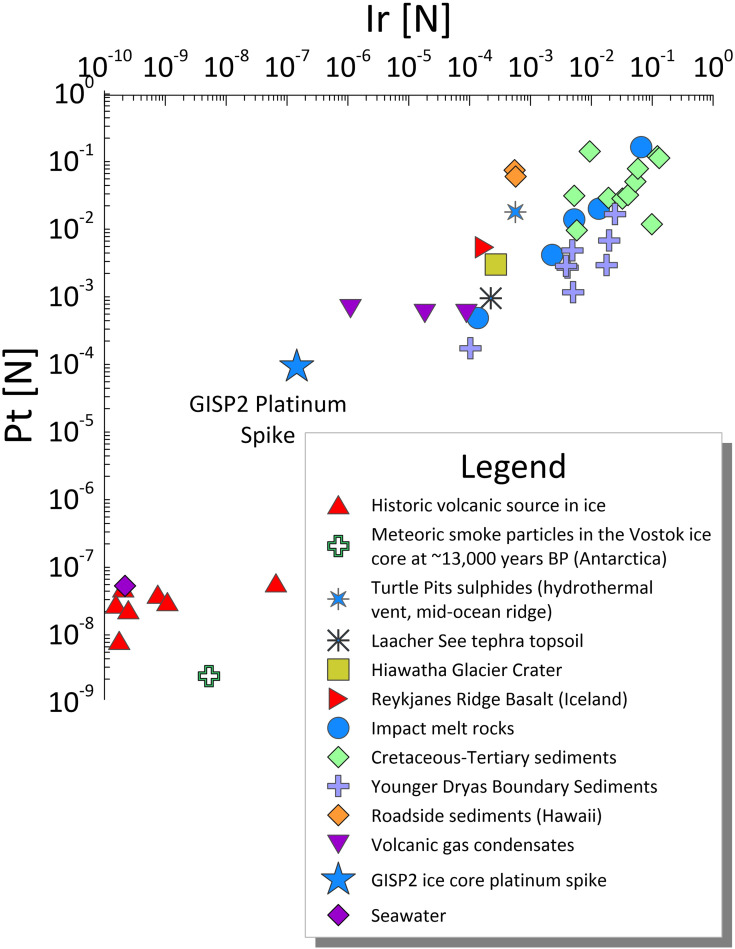
Geochemistry of relevant secondary sediments and deposits. Pt plotted against Ir normalised [N] against carbonaceous (Cl-type) chondrite, showing data from various sediments and ice sheets. Roadside sediments are from the Manoa and Palolo urban watersheds, Hawaii. Volcanic gas condensates from Kudryavy, Kurile Island Arc, Erta Ale, Ethiopia, Niuatahi-Motutahi, Tonga rear arc and Tolbachik, Kamchatka. The Turtle Pits sulphides are formed at a location along the Atlantic mid-ocean ridge. The Laacher See topsoil sample was measured in the study and was obtained above LST sampled at locality LST1. For fully referenced version of caption, please see SOM.

**Fig 6:**
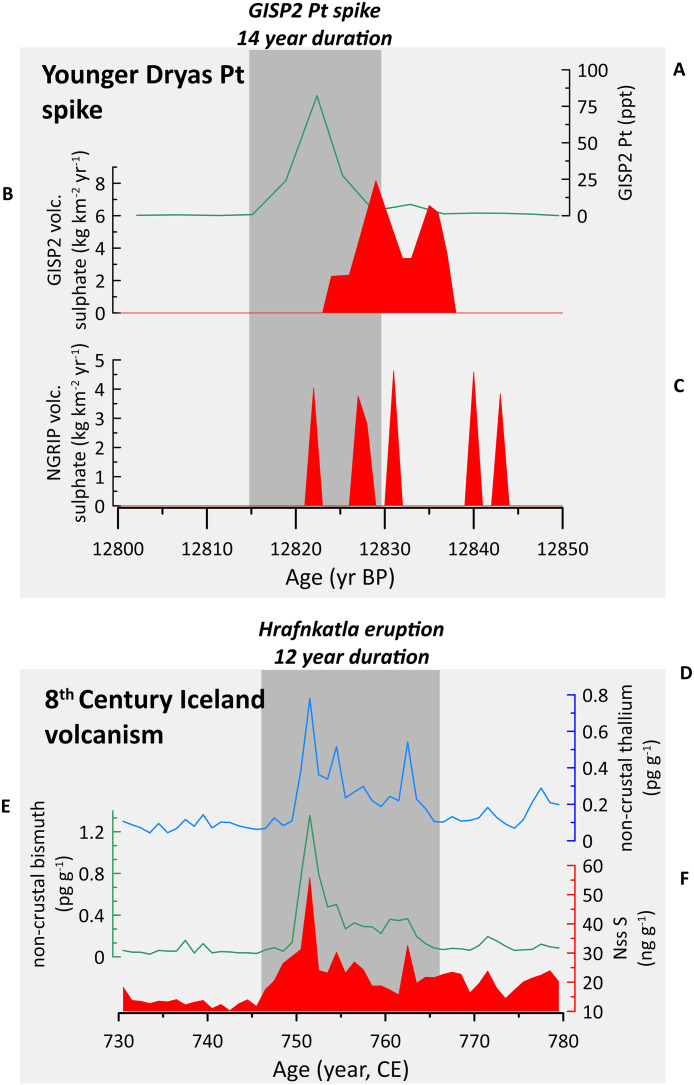
Greenland ice geochemistry across the Pt spike and known Iceland volcanic eruption intervals. A comparison of Greenland ice geochemistry across the Younger Dryas platinum spike (upper panels A-C) and the 8^th^ Century Katla eruption (bottom panels D-F). A) Platinum concentrations in the GISP2 ice core [40]; B) GISP2 volcanic sulphate concentrations [41,116]; C) NGRIP volcanic sulphate concentrations [41,116]; D) Mean non-crustal thallium concentrations from four Greenland ice cores [90]; E) Mean non-crustal bismuth concentrations from four Greenland ice cores [90]; F) Non-sea salt sulphur from the same four Greenland ice cores as in panels E and F [90].

This study generally corroborates Petaev et al. [40]’s interpretation that the ice geochemistry at the GISP2 Pt spike is dissimilar to common meteorites (such as chondrites) and magmas. To test if the Pt spike geochemistry could have resulted from fractionation following an impact or eruption, we compared its geochemistry to that of various sediments associated with impacts and eruptions (Fig 5). Notably, the GISP2 Pt anomaly differs from boundary sediments associated with the end-Cretaceous extinction (Fig 5) thought to have resulted from an extraterrestrial impact [67]. The most closely matching geochemical signature is that of volcanic gas condensates, particularly from the submarine volcanic complex Niuatahi-Motutahi (Tonga rear arc), suggesting that the Pt spike could reflect atmospheric fallout of Platinum Group Element (PGE)-fractionated volcanic aerosol (Fig 4). A subglacial Icelandic eruption proximal to the Greenland ice sheet would be most likely, but the 2022 Hunga Tonga-Hunga Ha’apai eruption demonstrated that volcanic plumes from submarine eruptions can reach phenomenal heights (57 km, well past the stratosphere and into at least the mesosphere) [68], so a more distal eruption is possible. Research has recently shown that Hunga-type submarine eruptions lose much of their sulphur via magma-seawater interactions while retaining their explosivity due to the vapourisation of seawater [69]. This introduces the possibility that an extremely explosive eruption might not leave a substantial sulphur signal in either Antarctic or Greenland ice. However, Icelandic eruptions are often long-lasting, heavy metal-rich fissure eruptions, which is compatible with the extended duration of the GISP2 Pt spike. Abbott et al. [41] note that a broad sulphate double spike exists in Greenland near the onset of the Pt spike in the GISP2 record (Fig 6), where the older spike (at 12,832 yr BP) coincides with a small sulphate signal in Antarctica but the younger spike (at 12,828 yr BP) does not. This broad double sulphate spike occurs right at the onset of the Pt spike and could reflect a long-lasting Icelandic fissure eruption. The Antarctic sulphate spike might represent an unknown southern hemisphere eruption occurring at the same time, or perhaps the older spike at 12,832 yr BP represents a bipolar eruption. A small volcanic sulphate spike is apparent within NGRIP that coincides with the peak Pt concentrations (Fig 6). Collectively, these data suggest that small eruptions did occur during the Pt spike interval, and we cannot rule out post-depositional diffusion of Pt within the ice to produce the Pt spike profile.

### Platinum in the volcanic system: melt

Given that our results discount the role of the LSE and also strongly argue against the role of a bolide impact as the cause of the GISP2 Pt anomaly, but are consistent with fractionated volcanic material, it is worth discussing a volcanic source further. PGEs tend to partition into an exsolved aqueous volatile phase [70,71]. Crystallisation of non-sulphur-bearing phases concentrates sulphur (S) species in melts, ultimately reaching saturation and precipitating non-volatile, sulphur-bearing phases [72]. In a sulphide (S^2-^)-rich melt, PGEs preferentially form covalent bonds with sulphide ions [73], thus becoming sequestered in sulphide liquids or alloys [74]. The sulphide liquid is either erupted as inclusions with the magma [e.g., 75] or may completely destabilize, liberating associated PGEs into the melt which results in sequestration (ore deposit formation) or outgassing into the atmosphere [72,73]. This destabilization mechanism is exacerbated during decompression because of increasing sulphur solubility and oxidation state [76]. Transport of sulphide and trace metals (in this case, PGEs) toward the top of the magma chamber may also result from rising vapour bubbles carrying sulphide melt droplets in a sulphide-melt saturated magma [77], increasing the likelihood of trace species outgassing. Ultimately, Pt is more volatile than Ir, consistent with the geochemistry of the GISP2 Pt spike (high Pt, low Ir). Platinum exsolves with other volatile species; for example, both Pt and Ir were enriched in volcanic gases compared with erupted basalt at Erta Ale (Ethiopia) by a factor of ~2,500 [78], and an even greater enrichment was observed (~7,500) at Tolbachik volcano (Kamchatka, Russia) [79].

Chloride- and sulphide-complexes both demonstrate the ability to transport Pt in volcanic systems [80,81]. Post-eruption, PGEs may remain associated with H_2_S in reducing conditions, however the oxidation and conversion of sulphur dioxide to sulphate in the volcanic aerosol results in loss of sulphide-complexes and thus perhaps PGE loss. This highlights the importance of hydrochloric acid as a major gas component [82] and its related chloride-complexes in volcanic aerosol for PGE transport. McConnell et al. [83] associated a small sulphate increase and a ~ 6x increase of chlorine (Cl) and volatile metal (e.g., lead, thallium) concentrations in the WAIS Divide and Byrd cores (Antarctica) with Cl-rich peralkaline eruptions of local Mount Takahe (West Antarctica) [71], illustrating a possible mechanism.

Here we suggest that Cl-rich volcanic aerosols from local eruptions may preferentially carry volatile trace metal cations and deposit them onto the Greenland ice sheet. Data from Kudryavy volcano (Kurile Volcanic Arc, Pacific Ocean) gas condensates show high Pt concentrations (0.49 ppb) compared to Ir (0.04 ppb) [80], and are also enriched in Cl (up to 15,380 ppm) [84], suggesting Cl-rich gases may have a role in transporting and concentrating Pt over Ir. Similarly, high Pt concentrations are found in gas emissions alongside high Cl contents at Tolbachik volcano (Kamchatka, Russia), with low Ir concentrations [79,85].

### A volcanic source for the GISP2 Pt spike

Volcanic-derived Pt is found in i) Greenland snow pits [55] and ii) Antarctic snow [56]. Therefore, it is conceivable that the GISP2 Pt spike simply reflects a volcanic deposit produced following PGE enrichment within a volcanic system or shortly following an eruption. Platinum and Ir concentrations in Reykjanes Ridge basalts [86] are orders of magnitude (60- and 1,000-fold, respectively) above the GISP2 Pt spike elemental concentrations and represent a plausible source to explain the GISP2 Pt spike geochemistry (Figs 4 and 5). The Icelandic plume has fed explosive volcanic activity in the North Atlantic since the Paleogene [87,88]. A recent study highlights that the 10^th^ Century Eldgjá eruption produced a cadmium spike in Greenland ice [89], and a similar study notes the presence of several spikes in bismuth and thallium in Greenland ice, coincident with known historical Icelandic eruptions [90]. In particular, the 8^th^ Century Hrafnkatla eruptive episode from 751–763 CE resulted in a ~ 12-year spike in both bismuth and thallium within four Greenland ice cores [90] (Fig 6), a very similar duration to the Pt spike (14 years). Although neither of these studies analysed platinum, the heavy metals cadmium, bismuth, and thallium are volatile metals transported and deposited as chloride aerosols, much like platinum. Many of Iceland’s volcanic zones were likely subglacial at the YDE onset and any emitted volcanic gases were potentially dissolved in meltwater, resulting in a weakened atmospheric volcanic sulphur signature [60,69]. For example, such dilution of trace constituents is observed following snowpack interaction at Tolbachik, Kamchatka [79]. A submarine eruption near Iceland is also possible, where the interaction of the eruption with seawater may have contribute further Cl to volcanic gases, as observed at Augustine volcano, Alaska [91], enhancing volatile trace metal cation transport. Finally, a comprehensive review of heavy metal deposition on the Antarctic Ice Sheet revealed that, over the Common Era, volcanism was responsible for 83–99% of the total pre-anthropogenic heavy metal contributions to the ice sheet [92], again highlighting an eruption as a likely source of the Pt spike.

Considering the above evidence, particularly: i) PGE behaviour in the magmatic system and atmosphere, ii) published Pt/Ir values for volcanic gas condensates, iii) known mechanisms for the removal of sulphur via water/magma interactions, iv) clear evidence for heavy metal enrichment Greenland ice following Icelandic fissure eruptions, and v) comprehensive evidence that volcanic eruptions are the main source of heavy metals deposited on ice sheets, it seems quite plausible that the elevated Pt concentrations in the GISP2 ice core centred around 12,822 yr BP are the result of a relatively small Icelandic fissure eruption, potentially interacting with melt or seawater. Quiescent degassing from local volcanoes or a long-lived effusive eruption could produce multiple Pt spikes, consistent with the gradually increasing Pt concentration preceding the highest concentrations of the GISP2 Pt spike. This perspective is also supported by probable increased melt generation rates caused by the unloading of the LGM ice sheet over Iceland during deglaciation, which led to average eruption rates up to 100 times higher than those over the past 5,000 years [93]. This is consistent with the major element chemistry of ash layers deposited on the Greenland Ice Sheet suggesting a predominantly Icelandic origin [60], and increased evidence of volcanism affecting Greenland during deglaciation [61,94–96]. The fractionation signature is consistent with a subglacial or submarine origin; interaction with water and/or salt could lead to removal of ash and SO_2_, which may explain the comparatively low sulphur values and lack of distinct tephra [97]. Regardless, some of the GISP2 samples with elevated Pt are coeval with the presence of volcanic sulphate within Greenland ice cores, though the overall shape of the Pt spike is not mimicked by the sulphate concentrations records.

### Implications for the Younger Dryas

**Fig 7:**
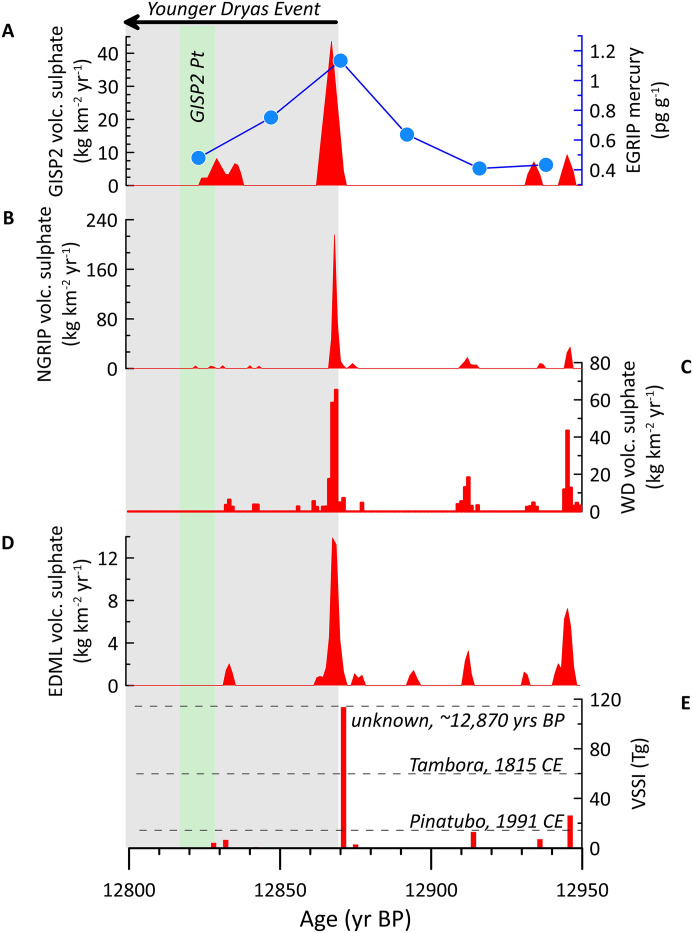
Ice core sulphate and mercury at the YDE onset. The sulphate spike at the onset of the Younger Dryas Event in four ice cores and the mercury spike in the East Greenland Ice-Core Project (EGRIP) core. A) Volcanic sulphate within the GISP2 ice core and mercury concentrations within the EGRIP ice core (Greenland) [63]; B) Volcanic sulphate within the NGRIP ice core (Greenland) [41,116]; C) Volcanic sulphate within the WAIS Divide (WD) ice core (Antarctica) [41,116]; D) Volcanic sulphate within the EDML ice core (Antarctica) [41,116]; E) Volcanic Stratospheric Sulphur Injection (VSSI) reconstructed for the Younger Dryas onset period [41,116]. The dashed lines represent the VSSI values associated with Tambora (1815 CE), Pinatubo (1991 CE), and the unknown ~12,870 yr BP eruption.

Large volcanic sulphate spikes at ~12,870 yr BP in the GISP2, NGRIP, WAIS Divide, and EPICA Dronning Maud Land (EDML) ice cores (Fig 7) are contemporaneous with the beginning of the δ^18^O decrease in Greenland ice cores and European temperature proxies associated with the onset of GS-1 [41,42,61] (Figs 2 and 3). Including other sulphate spikes in the ~century preceding the ~ 12,870 yr BP sulphur spike, the volcanic forcing preceding the YDE was calculated as exceeding any over the last 2,000 years [41]. Out of 1,113 eruptions identified from three Greenland ice cores using continuous flow analysis, the 12,978 yr BP and ~12,870 yr BP eruptions were ranked 16 and 17, respectively, in terms of amount of sulphur released and consequent negative climate forcing (−10.5 and −10.0 W m^-2^, respectively; for comparison, the 1991 CE Pinatubo eruption resulted in ~4 W m^-2^ radiative forcing [98]), and both eruptions probably occurred above 40ºN [61]. The juxtaposition of this intense, NH-specific volcanic forcing with the initiation of cooling into the YDE is remarkable (Figs 3 and 7).

Sulphur-rich eruptions create an atmospheric aerosol haze that advects around the globe, directly impacting the Earth’s radiative budget and indirectly influencing atmospheric circulation [6,99,100]. Volcanic sulphate aerosol injections cause net regional cooling, which may trigger a cascade of climate feedbacks such as sea ice growth, atmospheric circulation changes, and Atlantic Meridional Overturning Circulation weakening, all promoting more cooling [14,42,101–105]. Recent research suggests confinement of volcanic aerosols to one hemisphere results in an amplified radiative impact, and that northern hemisphere extratropical eruptions cause up to 80% more time-integrated radiative forcing than their tropical counterparts [106]. Therefore, the sulphur released during the inferred large, extratropical northern hemisphere ~12,870 yr BP eruption almost certainly formed a volcanic aerosol veil that wrapped around the northern hemisphere and, because the aerosols were largely restricted to one hemisphere, amplified its radiative impact [106,107]. The characteristics of the sulphate spike are consistent with the expected characteristics of a spike produced by the LSE, but the timing of the eruption is still debated [4,8,9,42,52,108,109], and in fact the hemispheric distribution of sulphate in earlier spikes referred to by [8] is also consistent with a predicted LSE signature. Consequently, the ~ 12,870 yr BP sulphur spike cannot currently be confidently linked to any specific eruption.

Regardless of a lack of definitive link to a specific volcano, the ~ 12,870 yr BP eruption occurred during a deglacial time interval with intermediate ice volume conditions, which accumulating evidence suggests could have triggered a positive long-term climate feedback [42,110,111]. This eruption was only one of a cluster of volcanic eruptions occurring before GS-1 that potentially contributed to cooling [41]. Our results suggest that explosive volcanism is a potentially straightforward explanation for the initial cooling that eventually led to the Younger Dryas cold event, consistent with previous works [41,42]. We conclude that the Pt spike was probably not associated with the YDE trigger as it occurred several decades after the event’s initiation, independently confirming recent results [39,41,57].

## Conclusions

Geochemical and chronological differences between the Laacher See eruption and the Pt spike near the beginning of the Younger Dryas event within the GISP2 ice core indicate that the LSE was not the source of the Pt spike. However, the geochemical signature of the GISP2 Pt spike is dissimilar to meteorites, magmas, other volcanic eruption signatures in snow and ice, and K-Pg sediments. The closest match is with highly fractionated volcanic condensates, and we suggest that a possible source of the GISP2 Pt spike at ~12,822 yr BP is a Cl- and Pt-rich local (Icelandic) volcanic eruption preferentially carrying Pt in chloro-complexes and depositing it onto the Greenland ice sheet, similar to the chloride-transported origin for volatile metals found in Antarctic ice cores associated with the 17.7 ka Mt Takahe eruptions [71], and across the last 2,000 years from volcanism in general [92]. The Pt spike may represent a diluted signal, perhaps from a submarine and/or subglacial volcano, which may have undergone further fractionation through atmospheric processing of the volcanic aerosol, though it is difficult to model this with the limited available data. Sulphur removal via sea or meltwater interactions with the magma could also explain the subdued volcanic sulphate signal in Greenland ice across the event. A volcanic origin for the Pt spike is consistent with observations strongly suggesting that Iceland volcanism experienced a peak in activity during deglaciation due to the unloading of glacial mass [60,93]. Additionally, the Pt anomaly lasted for over a decade, consistent with long-lasting, heavy metal-rich fissure eruptions characteristic of Iceland. We suggest that future work should prioritise: i) replicating the Pt signal in one or more of the other deep ice cores extracted from the Greenland Ice Sheet, ii) looking for other Pt concentration spikes across a wider time interval within the GISP2 ice core, and iii) measuring Pt concentrations across intervals characterised by known volcanic heavy metal enrichment (e.g., the 8^th^ Century Hrafnkatla eruption [90]). These tests would confirm whether the Pt concentration spike is apparent across Greenland and whether it reflects a unique event or one of many frequently recurring events. Additional work on the post-depositional mobility of Pt ions in ice would be beneficial in deciphering whether individual Pt peaks in ice cores represent individual eruptions, or whether Pt can ‘migrate’ to other sections of ice and not necessarily represent the primary deposition signal.

Our work independently supports recent results [39,41,57] that the Pt spike occurred ~50 years after the YDE’s initiation, so was not associated with its initial triggering mechanism. Because the geochemical signature of the Pt spike is most similar to volcanic condensates, we introduce the possibility that the Pt spike resulted from an unidentified volcanic eruption. The geochemical signature is consistent with a submarine or subglacial eruption, where interactions with water may have preferentially fractionated platinum into the gas phase and reduced the ash and sulphur content. Although we cannot rule out the impact of an unknown type of meteorite, the simplest explanation is that the Pt spike instead probably reflects a relatively small Icelandic fissure eruption. A fissure-style eruption, which are commonplace in Iceland, would also explain the 14-year-long duration of the Pt spike in the GISP2 ice core, an observation which is difficult to reconcile with an instantaneous impact of an extraterrestrial object. Furthermore, the YDE is just one of many rapid climate change events [1,38] intrinsic to intermediate climate states during transitions from glacial maxima to interglacials [42,112], and it is likely that these all shared similar origins, arguing against a rare cataclysmic trigger such as an impact. Our analysis suggests that the trigger for the Younger Dryas Event, and potentially these other rapid climate change events, was explosive volcanism, consistent with previous research [43,113].

## Supporting information

S1 File Supplementary text and tables. Supplemental material containing further details on materials and methods, an extended caption (with references) for Figure 4, Supplementary Tables (Table S1: Samples and descriptions; Table S2: Full table of geochemical results), and supplementary references.
(DOCX)
