# Supplementary material for: A possible volcanic origin for the Greenland ice core Pt anomaly near the Bølling-Allerød/Younger Dryas boundary
Source: PLoS One. 2025 Sep 18;20(9):e0331811. doi: 10.1371/journal.pone.0331811 (PMC12445550; doi:10.1371/journal.pone.0331811)
Supplement: S1 File — Supplemental material containing further details on materials and methods, an extended caption (with references) for Figure 4, Supplementary Tables (Table S1: Samples and descriptions; Table S2: Full table of geochemical results), and supplementary references. (DOCX) [file pone.0331811.s001.docx]

**Supporting Information for:**

The origin of a Greenland ice core geochemical anomaly near the Bølling-Allerød/Younger Dryas boundary

Charlotte E. Green^1,2^, James U.L. Baldini^2*^, Richard J. Brown^2^, Hans-Ulrich Schmincke^3^, Marie Edmonds^4^ and Thomas C. Meisel^5^

^1^Centre of Climate, Ocean and Atmosphere, Department of Earth Sciences, Royal Holloway, University of London, Egham, TW20 0EX, UK
^2^Department of Earth Sciences, University of Durham, Durham, DH1 3LE, UK
^3^GEOMAR Helmholtz Centre for Ocean Research, 24148 Kiel, Germany
^4^Department of Earth Sciences, University of Cambridge, Cambridge, CB2 3EQ, UK
^5^Chair of General and Analytical Chemistry, Montanuniversität Leoben, 8700 Leoben, Austria

*Department of Earth Sciences
Durham University
Durham, DH1 3LE
United Kingdom

**Email:**  james.baldini@durham.ac.uk

**This PDF file includes:**

Supplementary text

Tables S1 and S2

Supplementary figure caption

Supplementary References

Supplementary text

**Materials and methods**

**Materials**

The LSE produced three major tephra sequences: i) the Lower Laacher See Tephra (LLST); ii) the Middle Laacher See Tephra (MLST) and iii) the Upper Laacher See tephra (ULST), with marked changes in eruptive and depositional processes, lithology and geochemistry particularly in the ULST (Bogaard and Schmincke, 1985; Park and Schmincke, 2020a, b; Worner and Schmincke, 1984). Sulphur dioxide (SO_2_) released by the LSE in its Plinian phases (the LLST and MLST-B/C/D) (Park and Schmincke, 2020a, b) almost certainly led to severe Northern Hemisphere (NH) cooling, similar to that observed following other large explosive volcanic eruptions (Rampino and Self, 1984, 1992; Robock, 2000).

**Sample collection**

The face of the outcrop was cleared using a plastic spade, which was used to excavate approximately 10-20cm into the face of the outcrop in order to remove surface tephra that may have been contaminated by surface contamination, considering the location of the sites within quarried areas and the use of heavy machinery (excavators) with diesel engines. Catalytic converters in diesel engines are known to emit Pt and this could interfere with results (Soyol-Erdene et al., 2011). At least 1kg of each sample was obtained. For transport, samples were double bagged in plastic sample bags to reduce potential damage.

**Sample analysis**

**Fire assay + ICP-MS for Pt, Au, Pd**

A sample size of at least 50g was recommended, and this addresses a “nugget effect” that may occur with Pt distribution within a medium (e.g. Firestone et al., 2007; Moore et al., 2017). The sample is combined with fire assay fluxes (borax, soda ash, silica, litharge) and the mixture is placed in a fire clay crucible, with silver (Ag) included as a collector. The entire fusion process takes approximately 60 minutes, with the mixture preheated at 850°C, intermediate 950°C and finish 1060°C. The crucibles were extracted from the assay furnace; a lead button remaining at the bottom of the mould as the molten slag is poured from the crucible into a mould. The lead button is then positioned in a preheated cupel (cupelled at 950°C) which absorbs the lead and allows the Ag (doré bead) + Au, Pt and Pd to be recovered. To stop the Au, Pd and Pt from adsorbing onto the test tube, the Ag doré bead is digested in hot HNO_3_ + HCl at 95°C with a special complexing agent. A Perkin Elmer Sciex ELAN 6000, 6100 or 9000 ICP-MS was utilised following two hours of cooling to analyse for Au, Pt, and Pd. A tray of 42 samples included two method blanks, three sample duplicates, and two certified reference materials, and every 45 samples the ICP/MS was recalibrated. Smaller sample splits were utilised for high chromite or sulfide samples.

**Nickel sulfide fire assay INAA for Ir, Os, Ru, Rh**

Using HCl, the nickel sulfide button was dissolved, and the resulting residue containing all the PGE and Au was gathered on filter paper. Two irradiations and three separate counts were used on the residue to measure PGE and Au. One batch of 34 samples included two blanks, three certified standards and three duplicates. The INAA analytical technique measures gamma radiation induced in the sample by irradiation with neutrons which were sourced from a nuclear reactor. A “fingerprint” of gamma radiation was released by each element which was then measured and quantified.

**Lithium metaborate/tetraborate Fusion ICP Whole Rock and ICP-MS for major and trace elements**

A batch system was used, with each batch containing a method reagent blank, certified reference material and 17% replicates. Samples were fused in an induction furnace following mixing with a lithium metaborate and lithium tetraborate flux. A 5% nitric acid solution containing an internal standard was instantly mixed with the molten melt until the melt was completely dissolved (~30 minutes). Using a combination simultaneous/sequential Thermo Jarrell-Ash ENVIRO II ICP or a Varian Vista 735 ICP, major oxides and selected trace elements were analysed in the samples. Seven prepared USGS and CANMET certified reference materials were used for calibration. For every group of ten samples, one of the seven standards was utilised during analysis. Following these analyses, samples were scanned for base metals. Low totals resulted in re-fusion and reanalysis of the samples. A Perkin Elmer Sciex ELAN 6000, 6100, or 9000 ICP-MS were used for analysis after dilution of the fused sample. Per group of samples, three blanks and five controls (three before sample group and two after) were analysed, and every 15 samples the duplicates were fused and analysed. The instrument was recalibrated every 40 samples.

**Supplementary discussion**

**Mobility of the PGEs in snow and ice**

Post-depositional mobility of the PGEs (particularly Ir) in marine sediments, where elements are redistributed by variations in redox processes, may result in small peaks in concentrations and offset PGE ratios (Colodner et al., 1992). However, post-depositional mobility of the PGEs is not well studied in snow and ice. Air-snow transfer and post-depositional modification may act to distort or filter the atmospheric signal of volcanic aerosol fallout to some degree prior to preservation in the ice core (Bales et al., 1992), but the extent to which is unclear.

**Supplementary figure caption**

Below is the figure caption for Figure 4 in the main text with full citations. It was decided to not include the citations in the caption in the main text to reduce the number of citations in the bibliography. The caption is otherwise identical to the one in the main text.

**Fig 4.** **Geochemistry of relevant primary materials.** Pt (ppb) plotted against Ir (ppb) normalised [N] against Cl chondrite (McDonough and Sun, 1995), showing Pt/Ir ratios of i) historic volcanic sources in ice, represented by triangles and the orange field (Gabrielli et al., 2008; Soyol-Erdene et al., 2011); ii) YDB sediments in North America and Europe, represented by crosses and the pink field (Andronikov and Andronikova, 2016; Firestone et al., 2007; Moore et al., 2017; Paquay et al., 2009); iii) Cretaceous-Tertiary (Cretaceous-Paleogene) boundary (K-Pg) sediments, represented by stars and the green field (Evans et al., 1993a); iv) impact melt rocks in craters created by bolide impacts, represented by circles and the blue field (Evans et al., 1993b; Tagle and Claeys, 2004); v) Hiawatha Glacier crater (HGC) (Kjaer et al., 2018); vi) roadside sediments from the Manoa and Palolo urban watersheds, Hawaii (Sutherland et al., 2007), represented by grey crosses; vii) seawater represented by the purple diamond (Nozaki, 1997); viii) volcanic gas condensates from Kudryavy, Kurile Island Arc (Yudovskaya et al., 2008), Erta Ale, Ethiopia (Zelenski et al., 2013), Niuatahi-Motutahi, Tonga rear arc (Park et al., 2016) and Tolbachik, Kamchatka (Chaplygin et al., 2016), represented by inverted purple triangles and the purple field; ix) Reykjanes Ridge basalt (RRB) (Schilling and Kingsley, 2017), x) Turtle Pits sulphides (TPS) (Pasava et al., 2007); xi) meteoric smoke particles in the Vostok ice core, Antarctica (MSPV) at ~13,000 years BP (Gabrielli et al., 2008); xii) LST TS – Topsoil overlying the LST from locality LST1 (this study) and xiii) the GISP2 Pt spike (GISP2) (Petaev et al., 2013).

**Supplementary Tables**

| \| **Locality** \| **Co-ordinates** \| **LST Unit** \| **Samples** \| **Brief Description** \| \| --- \| --- \| --- \| --- \| --- \| \| LST1 \| 50°24’38” N, 7°22’31” E \| Lower Laacher See Tephra (LLST) \| LST001, LST001A, LST002, LST002A, LST003, LST003A, LST003B \| Pyroclastic fall deposit. Fine grained pumic lapilli with 2-5% fine grained lithic clasts (Devonian slate xenolith). Sharp boundary to 'Big Bang' layer of fine to coarse grained pumice lapilli with 30-50% coarse grained lithic clasts (Devonian slate xenoliths). Thin ash layer above. \| \| LST2 \| 50°24'20 "N, 7°19'31.63"E \| Middle Laacher See Tephra (MLST) \| LST004, LST,005, LST006, LST007, LST007A \| Pyroclastic fall deposit. Alternating ash layers and pumice layers with fine to coarse grained pumice lapilli and 30% fine to coarse slate xenoliths. Lithic content decreases up through the pumice-rich layers. Sharp boundary marking transition to a large pumice bed with pumice lapilli and 10-30% slate xenolith clasts. Pumice lapilli becoming more rounded toward the top of the outcrop. Grey, denser and more phenocryst-rich pumice lapilli present near the top of the outcrop before transitioning into soil with rare pumice and lithic clasts. \| \| LST3 \| 50°23’30” N, 7°16’23” E \| Upper Laacher See Tephra (ULST) \| LST008 \| Pyroclastic density current deposit. Interbedded coarse grained pumice lapilli rich layers with ash layers and dune-bedded layers representing filled channels. The middle of the outcrop is dominated by coarse grained clast rich layers with mixed lithologies of pumice lapilli (phenocryst rich), cumulate and plutonic rock fragments and slate xenoliths. The upper outcrop comprises laminated fine and coarse flow layers with some thin ash layers and blocks or bombs (some >50cm) with associated impact craters. \| |  |  |  |  |
| --- | --- | --- | --- | --- | --- | --- | --- | --- | --- | --- | --- | --- | --- | --- | --- | --- | --- | --- | --- | --- | --- | --- | --- | --- |

**Table S1: Samples and descriptions**

| **Sample no.** | **Pt (ppb)** | **Ir (ppb)** | **Hf (ppm)** | **Lu (ppm)** | **Al2O3 (wt%)** |
| --- | --- | --- | --- | --- | --- |
| LST 001 | 0.2 | < 0.1 | 37 | 1.13 | 20.59 |
| LST 001A | 0.1 | < 0.1 | 25 | 0.88 | 17.65 |
| LST 002 | < 0.1 | < 0.1 | 35.8 | 1.06 | 21.09 |
| LST 002A | 0.1 | 0.2 | 23.6 | 0.81 | 16.88 |
| LST 003 | < 0.1 | < 0.1 | 31.3 | 1.05 | 20.68 |
| LST 003A | 0.2 | < 0.1 | 19.2 | 0.72 | 17.08 |
| LST 003B | 0.4 | < 0.1 | 5.1 | 0.43 | 14.52 |
| LST 004 | < 0.1 | < 0.1 | 14.8 | 0.55 | 20.6 |
| LST 005 | < 0.1 | < 0.1 | 14.4 | 0.55 | 20.51 |
| LST 006 | < 0.1 | < 0.1 | 11.3 | 0.47 | 20.08 |
| LST 007 | < 0.1 | < 0.1 | 7.6 | 0.35 | 19.19 |
| LST 007A | < 0.1 | < 0.1 | 9.4 | 0.4 | 20.01 |
| LST 008 | < 0.1 | < 0.1 | 4.6 | 0.29 | 17.92 |
| SAMPLE A | < 0.1 | < 0.1 | 30.8 | 0.98 | 21.37 |
| SAMPLE B | < 0.1 | < 0.1 | 7.8 | 0.35 | 19.86 |
| SAMPLE C | 0.1 | < 0.1 | 14.3 | 0.52 | 20.37 |
| SAMPLE D | < 0.1 | < 0.1 | 4.9 | 0.31 | 18.75 |
| LST1 TS | 0.9 | < 0.1 | 7.5 | 0.43 | 17.79 |

*Table S2: Full table of ActLabs results*

**Supplementary references**

Andronikov, A.V., Andronikova, I.E., 2016. Sediments from around the Lower Younger Dryas Boundary (Se Arizona, USA): Implications from La-Icp-Ms Multi-Element Analysis. Geogr Ann A 98, 221-236.

Bales, R., Dibb, J., Neftel, A., 1992. The GISP2 ice core and snow‐atmosphere chemical exchange. Eos, Transactions American Geophysical Union 73, 213.

Chaplygin, I.V., Lavrushin, V.Y., Dubinina, E.O., Bychkova, Y.V., Inguaggiato, S., Yudovskaya, M.A., 2016. Geochemistry of volcanic gas at the 2012-13 New Tolbachik eruption, Kamchatka. J Volcanol Geoth Res 323, 186-193.

Colodner, D.C., Boyle, E.A., Edmond, J.M., Thomson, J., 1992. Postdepositional Mobility of Platinum, Iridium and Rhenium in Marine-Sediments. Nature 358, 402-404.

Evans, N.J., Gregoire, D.C., Goodfellow, W.D., Mcinnes, B.I., Miles, N., Veizer, J., 1993a. Ru/Ir Ratios at the Cretaceous-Tertiary Boundary - Implications for Pge Source and Fractionation within the Ejecta Cloud. Geochim Cosmochim Ac 57, 3149-3158.

Evans, N.J., Gregoire, D.C., Grieve, R.A.F., Goodfellow, W.D., Veizer, J., 1993b. Use of Platinum-Group Elements for Impactor Identification - Terrestrial Impact Craters and Cretaceous-Tertiary Boundary. Geochim Cosmochim Ac 57, 3737-3748.

Firestone, R.B., West, A., Kennett, J.P., Becker, L., Bunch, T.E., Revay, Z.S., Schultz, P.H., Belgya, T., Kennett, D.J., Erlandson, J.M., Dickenson, O.J., Goodyear, A.C., Harris, R.S., Howard, G.A., Kloosterman, J.B., Lechler, P., Mayewski, P.A., Montgomery, J., Poreda, R., Darrah, T., Hee, S.S.Q., Smitha, A.R., Stich, A., Topping, W., Wittke, J.H., Wolbach, W.S., 2007. Evidence for an extraterrestrial impact 12,900 years ago that contributed to the megafaunal extinctions and the Younger Dryas cooling. P Natl Acad Sci USA 104, 16016-16021.

Gabrielli, P., Barbante, C., Plane, J.M.C., Boutron, C.F., Jaffrezo, J.L., Mather, T.A., Stenni, B., Gaspari, V., Cozzi, G., Ferrari, C., Cescon, P., 2008. Siderophile metal fallout to Greenland from the 1991 winter eruption of Hekla (Iceland) and during the global atmospheric perturbation of Pinatubo. Chem Geol 255, 78-86.

Kjaer, K.H., Larsen, N.K., Binder, T., Bjork, A.A., Eisen, O., Fahnestock, M.A., Funder, S., Garde, A.A., Haack, H., Helm, V., Houmark-Nielsen, M., Kjeldsen, K.K., Khan, S.A., Machguth, H., McDonald, I., Morlighem, M., Mouginot, J., Paden, J.D., Waight, T.E., Weikusat, C., Willerslev, E., MacGregor, J.A., 2018. A large impact crater beneath Hiawatha Glacier in northwest Greenland. Sci Adv 4.

McDonough, W.F., Sun, S.S., 1995. The composition of the Earth. Chem Geol 120, 223-253.

Moore, C.R., West, A., LeCompte, M.A., Brooks, M.J., Daniel Jr, I.R., Goodyear, A.C., Ferguson, T.A., Ivester, A.H., Feathers, J.K., Kennett, J.P., Tankersley, K.B., Adedeji, A.V., Bunch, T.E., 2017. Widespread platinum anomaly documented at the Younger Dryas onset in North American sedimentary sequences. Sci Rep-Uk 7, 44031.

Nozaki, Y., 1997. A fresh look at element distribution in the North Pacific Ocean. Eos, Transactions American Geophysical Union.

Paquay, F.S., Goderis, S., Ravizza, G., Vanhaecke, F., Boyd, M., Surovell, T.A., Holliday, V.T., Haynes, C.V., Claeys, P., 2009. Absence of geochemical evidence for an impact event at the Bolling-Allerod/Younger Dryas transition. P Natl Acad Sci USA 106, 21505-21510.

Park, J.W., Campbell, I.H., Kim, J., 2016. Abundances of platinum group elements in native sulfur condensates from the Niuatahi-Motutahi submarine volcano, Tonga rear arc: Implications for PGE mineralization in porphyry deposits. Geochim Cosmochim Ac 174, 236-246.

Pasava, J., Vymazalová, A., Petersen, S., 2007. PGE fractionation in seafloor hydrothermal systems:: examples from mafic- and ultramafic-hosted hydrothermal fields at the slow-spreading Mid-Atlantic Ridge. Miner Deposita 42, 423-431.

Petaev, M.I., Huang, S.C., Jacobsen, S.B., Zindler, A., 2013. Large Pt anomaly in the Greenland ice core points to a cataclysm at the onset of Younger Dryas. P Natl Acad Sci USA 110, 12917-12920.

Schilling, J.-G., Kingsley, R., 2017. Platinum-group elements (PGE), Re, Ni, Cu, Ag and Cd variations along the Reykjanes Ridge and Iceland South-West Neovolcanic Rift Zone, from 50°N to 65°N: Implications on sulfide bearing PGE mantle source heterogeneities and partial melting effects. Interdisciplinary Earth Data Alliance (IEDA).

Soyol-Erdene, T.O., Huh, Y., Hong, S., Do Hur, S., 2011. A 50-Year Record of Platinum, Iridium, and Rhodium in Antarctic Snow: Volcanic and Anthropogenic Sources. Environ Sci Technol 45, 5929-5935.

Sutherland, R.A., Pearson, D.G., Ottley, C.J., 2007. Platinum-group elements (Ir, Pd, Pt and Rh) in road-deposited sediments in two urban watersheds, Hawaii. Appl Geochem 22, 1485-1501.

Tagle, R., Claeys, P., 2004. Comet or asteroid shower in the late Eocene? Science 305, 492-492.

Yudovskaya, M.A., Tessalina, S., Distler, V.V., Chaplygin, I.V., Chugaev, A.V., Dikov, Y.P., 2008. Behavior of highly-siderophile elements during magma degassing: A case study at the Kudryavy volcano. Chem Geol 248, 318-341.

Zelenski, M.E., Fischer, T.P., de Moor, J.M., Marty, B., Zimmermann, L., Ayalew, D., Nekrasov, A.N., Karandashev, V.K., 2013. Trace elements in the gas emissions from the Erta Ale volcano, Afar, Ethiopia. Chem Geol 357, 95-116.
